# MYB-CC transcription factor, *TaMYBsm3*, cloned from wheat is involved in drought tolerance

**DOI:** 10.1186/s12870-019-1751-9

**Published:** 2019-04-15

**Authors:** Yaqing Li, Shichang Zhang, Nan Zhang, Wenying Zhang, Mengjun Li, Binhui Liu, Zhanliang Shi

**Affiliations:** 1 Shijiazhuang Academy of Agriculture and Forestry Sciences, No.479 Shengli North Street, Chang’an district, Shijiazhuang, 050041 Hebei Province China; 2 Dryland Farming Institute, Hebei Academy of Agriculture and Forestry Sciences, Hengshui, 053000 China

**Keywords:** Drought stress, *TaMYBsm3*, Transcription factor, *Triticum aestivum* L, *TaMYBsm3-D* transgenic *Arabidopsis*, Validation

## Abstract

**Background:**

MYB-CC transcription factors (TFs) genes have been demonstrated to be involved in the response to inorganic phosphate (Pi) starvation and regulate some Pi-starvation-inducible genes. However, their role in drought stress has not been investigated in bread wheat. In this study, the *TaMYBsm3* genes, including *TaMYBsm3-A*, *TaMYBsm3-B*, and *TaMYBsm3-D*, encoding MYB-CC TF proteins in bread wheat, were isolated to investigate the possible molecular mechanisms related to drought-tolerance in plants.

**Results:**

*TaMYBsm3-A*, *TaMYBsm3*-*B*, and *TaMYBsm3*-*D* were mapped on chromosomes 6A, 6B, and 6D in wheat, respectively. *TaMYBsm3* genes belonged to MYB-CC TFs, containing a conserved MYB DNA-binding domain and a conserved coiled–coil domain. *TaMYBsm3-D* was localized in the nucleus, and the N-terminal region was a transcriptional activation domain. T*aMYBsm3* genes were ubiquitously expressed in different tissues of wheat, and especially highly expressed in the stamen and pistil. Under drought stress, transgenic plants exhibited milder wilting symptoms, higher germination rates, higher proline content, and lower MDA content comparing with the wild type plants. *P5CS1*, *DREB2A*, and *RD29A* had significantly higher expression in transgenic plants than in wild type plants.

**Conclusion:**

*TaMYBsm3-A*, *TaMYBsm3*-*B*, and *TaMYBsm3*-*D* were associated with enhanced drought tolerance in bread wheat. Overexpression of TaMYBsm3-D increases the drought tolerance of transgenic *Arabidopsis* through up-regulating *P5CS1*, *DREB2A*, and *RD29A*.

## Background

Drought is a severe environmental stress influencing the productivity of corps worldwide. Drought tolerance is a complex phenotype in plants, and is associated with diverse metabolic and morphological pathways [1]. Transcriptional regulation contributes to the response of drought stress in plants [2]. It is noteworthy that the transcription factors (TFs) function in the regulation of diverse physiological and biochemical responses to drought stress by activating diverse stress-related genes [3]. Until now, various TFs have been identified to be associated with the drought stress response, including MYB, bZIP, AP2/ERF, NAC, and WRKY [4, 5]. The MYB TFs comprise a larg gene family in plants [6].

MYB, firstly discovered in maize, is characterized by containing a MYB DNA-binding domain in its N-terminus that consists of one or more imperfect tandem repeat(s) [7, 8]. The MYB TFs participate in many development processes in plants, such as regulation of cell cycle and cell development, controlling of primary and secondary metabolism, and responses to abiotic and biotic stresses [9, 10]. To date, massive MYB proteins have been detected in diverse plants, such as rice, wheat, *Arabidopsis*, grapes, and petunias [11]. It has been reported that PbrMYB21, an MYB TF of *Pyrus betulaefolia*, can promote drought tolerance by modulating polyamine levels via arginine decarboxylase genes [12]. The up-regulated OsMYB48–1, an MYB TF of *Oryza sativa*, promotes drought tolerance of rice by influencing the synthesis of ABA [13]. Overexpression of TaODORANT1, an R2R3-type MYB TF of *Triticum aestivum*, promotes drought tolerance of tobacco by activating genes associated with stress and reactive oxygen species (ROS) [14]. The molecular mechanisms underlying MYB proteins functioning on drought tolerance of plants have been intensively studied. A previous study showed that overexpressed abscisic acid (ABA)- and drought-inducible AtMYB96 could activate the wax biosynthesis genes and increase the deposition of cuticular waxes, thereby leading to enhenced drought tolerance in *Arabidopsis* [15]. Moreover, a recent study reported that the TaMYB31s were upregulated under ABA treatment, and overexpressed TaMYB31-B could increase ABA sensitivity during seed germination [16]. Specially, ABA regulates adaptive responses to environmental stresses, and promotes the tolerance to various abiotic stresses including drought [17]. The balance between ABA biosynthesis and catabolism determines its role under drought-stress condition [18]. Thus, the increased drought tolerance are partly attributed to ABA [16].

MYB-CC TFs are the members of the MYB TF superfamily, being characterized by containing a conserved MYB DNA-binding domain and a coiled–coil (CC) domain, which is a potential dimerization motif. [19]. Currently, MYB-CC TFs are demonstrated to be related to the response to inorganic phosphate (Pi) starvation and regulate a series of Pi-starvation-inducible genes [20–22]. Nevertheless, its role in drought tolerance has not been reported to our knowledge. Bread wheat (*Triticum aestivum* L.) is a main food crop in the world. The repercussions of climate change, particularly drought stress, severely affect wheat yield globally. Identification of drought-responsive genes, especially TF genes, may be of benefit by revealing the molecular mechanisms involved in drought responses in plants. Presently, many wheat MYB genes containing full-length gene sequences have been isolated [23], including some drought stress response-associated gene, such as *TaMYB31* [16]. Given the drought tolerance of MYB genes in wheat and other plants, we hypothesized that MYB-CC TF genes may also play a role in drought stress in bread wheat.

Herein, three *TaMYBsm3* homologue genes (*TaMYBsm3-A*, *TaMYBsm3-B*, and *TaMYBsm3-D*) isolated from wheat were cloned and characterized by screening bacterial artificial chromosomal (BAC) library. The ability of *TaMYBsm3* genes in regulating the drought response was investigated in *TaMYBsm3-D* transgenic *Arabidopsis*, due to the high conservation of *TaMYBsm3-D* in wheat. We for the first time revealed that TaMYBsm3, a MYB-CC TF, was involved in plant drought stress response. Our results may shed light on the molecular mechanism of drought response and improve the cultivation of plant varieties with drought tolerance.

## Methods

### Cloning of *TaMYBsm3* homologue genes

Nested PCR primers (NP), as shown in Table 1, were designed to screen BAC libraries [24] of *Triticum aestivum* (*T. aestivum*) cultivar Shimai 15 that contained *TaMYBsm3* genes according to the expressed sequence tags (EST) (GenBank No. BJ243280). BAC pool plasmids served as template and the PCR programs included 35 cycles of 94 °C for 45 s, 55 °C for 45 s, 72 °C for 45 s, and an extension at 72 °C for 5 min. Then three BACs containing *TaMYBsm3* genes were used to construct 10-kb subclone libraries using the *BamHI* site of the pCC1BAC vector. Three full-length genomic DNA sequences of *TaMYBm3* genes were isolated by screening subclone libraries and sequencing subclones. Full-length cDNAs were amplified from a cDNA template of 2-week-old Shimai15 seedlings. The primers (TaMYBsm3-AFL, TaMYBsm3-BFL, and TaMYBsm3-DFL) (Table 1) were used to isolate the full-length cDNAs and genomic DNAs of the *TaMYBsm3* genes were designed on the basis of the *TaMYBsm3* genomic DNA sequences. The PCR and RT-PCR were amplified using 2 × Pfu PCR MasterMix (TIANGEN BIOTECH, Beijing, China). The PCR program was 95 °C for 5 min, 30 cycles of 94 °C 30 s, 55 °C for 30 s, 72 °C for 3–5 min and then at 72 °C for 8 min.

**Table 1:** Specific primer sequences

Primer name	Upstream primer (5′-3′)	Downstream primer (5′-3′)
NP	5’CAATGCCATGGATTTCAGTG 3’	5′ GCCATCACCGACAACGAGG 3’
	5′ GCTCTTTACGTGAAAGATTGTC 3’	5′ CTTCAGCACACCCTTGGGAG 3’
TaMYBsm3-AFL	5’GCTTCTGGACGAACACGG3’	5’CACTAGAACTCTACATGGCAAG3’
TaMYBsm3-BFL	5’GCTTCTGGACGAACACGG3’	5’CACTAGAACTCTACATGGCAAG3’
TaMYBsm3-DFL	5’GACTGGCGGCTGATTTGG3’	5’CACTAGAACTCTACATGGCAAG3’
DW	5’GAGCAACTAGAGGTAACAGTGT3’	5’CTGGCCTTCTGTTGTTCTAG3’
TR1	5’CGGGATCCATGAGCACACAGAGTG3’	5’TGCACTGCAGCTATTCGGTGTCTG3’
TR2	5’CGGGATCCATGAGCACACAGAGTG3’	5’TGCACTGCAGGAAATCCATGGCATTG3’
TR3	5’CGGGATCCATGAGTGAGCAGCTGGAG3’	5’TGCACTGCAGCTATTCGGTGTCTG3’
TR4	5’CGGGATCCATGTCACCTAGTCTAGTATC3’	5’TGCACTGCAGCTTCTTGTTGCCAG3’
TSR	5’GCTCTAGAATGAGCACACAGAGTGTA3’	5’CGGGATCCCGGTTCACTTTCA AGATC3’
WT	5’AGGATACTTGGCAAACAAACGA3’	5’CAATGGCTTCTACGAGACCGA3’
WQA	5’ACTAGAGGTGCAAAGGAAATTACAA3’	5’AAGTTCTGTTCTATTTCGGTGTCG3’
WQB	5’TGAGTTCATCACCATCATCATCG3’	5’TTTGCCAGAATCAATTCCAGG3’
WQD	5’GAGCAACTAGAGGTGCAAAGGG3’	5’GTTTTCTCCTCTGGTTTCGGC3’
TaMYBsm3R	5’CGGGATCCGACTGGCGGCTGATTTGG3’	5’TGCACTGCAGCACTAGAACTCTATGGCAAG3’
DREB2A	5’CTGGAGAATGGTGCGGAAGA3’	5’CAGATAGCGAATCCTGCTGTTGT3’
P5CS1	5’GCGCATAGTTTCTGATGCAA3’	5’TGCAACTTCGTGATCCTCTG3’
RD29A	5’ATCACTTGGCTCCACTGTTGTTC3’	5’ACAAAACACACATAAACATCCAAAGT3’
Actin	5’TCGCTGACCGTATGAGCAAAG3’	5’TGTGAACGATTCCTGGACCTG3’

A set of nullisomic-tetrasomic lines of *T. aestivum* ‘Chinese Spring’ (CS NT) were used for determining the chromosomal location of the TaMYBsm3 homologue genes. Primers used in the chromosomal location of *TaMYBsm3* genes (DW) (Table 1) were designed in accordance with the differences in DNA sequence. DNA isolated form a nullisomic tetrasomic line of Chinese Spring (NT-CS) was used as the template. The PCR program included 30 cycles of 94 °C for 30 s, 60 °C for 30 s, 72 °C for 60 s, and an extension at 72 °C for 8 min.

### Sequence analysis

Sequence assembly as well as coding region prediction were conducted with Lasergene SeqMan II Module (DNAStar). Phylogenetic analysis was carried out using neighbor-joining and maximum parsimony methods with 1000 bootstrap replicates using MEGA4.1, and a phylogenetic tree was constructed based on 20 MYBsm3-like proteins (including TaMYBsm3 proteins). In order to make stronger phylogenetic inferences and to test whether they were congruent, we reconstructed trees by Bayesian analysis using MrBayes v. 3.2.2 [× 64]. Multiple sequence alignment was performed using the software of ClustalW1.83.

### Transactivation assay *of TaMYBsm3-D in yeast*

A transactivation assay was performed as previously described [25]. The full-length cDNA (TaMYBsm3F), N-terminal region (TaMYBsm3N, 1-148aa), C-terminal region (TaMYBsm3C, 149-386aa), and myb-SHAQKYF region (TaMYBsm3M, 184-308aa) of *TaMYBsm3-D* were amplified using specific primers (TR1-TR4) (Table 1). After being cloned in the pMD18-T plasmid (TaKaRa, Dalian, China), the PCR products were integrated into the GAL4 DNA-binding domain of the pGBKT7 plasmid (Clontech, Shanghai, China) between the *Bam*HI and *Pst*I sites. Then, the recombinants were transformed into yeast strain Y190 (MATa, HIS3, lacZ, rp1, leu2, cyhr2). Next, they were cultured in selection medium (SD/−Trp/−His/−Ade) containing 25 mM 3-amino-1, 2, aminotriazole for 1 day, the β-galactosidase activity was detected by X-gal staining [26].

### Subcellular localization assay of the TaMYBsm3-D-GFP fusion protein

The amplification of full-length cDNA sequence of *TaMYBsm3*-*D* was performed by PCR using specific primers (TSR) (Table 1) and inserted into the binary vector pCAMBIA1300-sGFP between the sites of *Xba*I and *BamH*I. Positive clones were identified by sequencing. The construct was transformed into epidermal cells of *Nicotiana benthamiana* (*N. benthamiana*) in accordance with particle bombardment technology. The fluorescence of GFP was observed under a microscope (Leica SP8, Germany).

### Real time-quantitative PCR (RT-qPCR)

Total RNA was extracted from different tissues (root, stem, leaf, stamen, pistil, and seed) of wild type (WT) plants (Shimai 15) using TRIZOL reagent. Synthesis of first-strand cDNA was conducted using a cDNA Synthesis Kit (TaKaRa). RT-qPCR was performed to detect the expression pattern of *TaMYBsm3*-*A*, *TaMYBsm3*-*B*, and *TaMYBsm3*-*D* using specific primers (WT, WQA, WQB, and WQD) (Table 1). The PCR program included 40 cycles of 95 °C for 30 s, 60 °C for 30 s, and 72 °C for 30 s. The *β-actin* gene served as an internal control. The amount of mRNA relative to *β-actin* was calculated using the 2^-△△Ct^ method.

In addition, WT plants were treated with 16% polyethylene glycol (PEG) 8000, and the expression of *TaMYBsm3*-*A*, *TaMYBsm3*-*B*, and *TaMYBsm3*-*D* was detected in leaves and roots at different time points.

### Construction of transgenic plants with *TaMYBsm3-D*

The open reading frame (ORF) of *TaMYBsm3-D* was amplified with specific primers (TaMYBsm3R) (Table 1) and inserted into the vector pCAMBIA2300 containing the CaMV35S promoter between the *BamH*I and *Pst*I sites. The resultant vector was named CaMV35s:*TaMYBsm3-D*. Then, CaMV35s:*TaMYBsm3-D* was transformed into *Arabidopsis thaliana* cultivar Columbia-0 using the *A. tumefaciens* strain GV3101 in accordance with the floral dipping method [27]. Three homozygous transgenic lines (L4, L8, and L13) were confirmed by RT-qPCR and isolated for further assays. In addition, WT plants were treated with 16% PEG8000, and the expression of *TaMYBsm3*-*A*, *TaMYBsm3*-*B*, and *TaMYBsm3*-*D* was detected in leaves and roots at different time points.

### Drought stress assay

#### Phenotypic observation

The transgenic plants together with WT plants (*N* = 30) were grown for 4 weeks under normal conditions. Then, watering was stopped for 2 weeks. To investigate drought adaptability, plants were re-watered for 2 days, and the phenotypic changes were photographed at different time points. There were three experimental replicates.

#### Germination assay

The seeds of the WT and transgenic plants (*N* = 100) were planted in 1/2 MS medium that contained 16% PEG8000. Seeds with radical tip expanding the seed coat were considered as germination. The number of germinated seeds was recorded, and the germination rate was calculated. There were three experimental replicates.

#### Water-loss assay

The WT plants and transgenic plants (*N* = 30) were grown for 4 weeks under normal conditions. The fresh weight of aerial parts was recorded every hour until no obvious difference was detected between two adjacent time points. After 24 h of drying at 85 °C, the dry weight was measured. The water-loss rate was calculated as: (fresh weight at different time points - dry weight) / (initial fresh weight - dry weight). There were three experimental replicates.

#### Proline and malondialdehyde (MDA) detection

The transgenic plants together with WT plants (N = 30) were grown for 4 weeks under normal conditions. After 2 days of drought treatment, rosette leaves were collected. The proline content (nmol g^− 1^ FW) was measured in accordance with the ninhydrin acid reagent method. MDA content was measured as previously described [28]. There were three experimental replicates.

#### Expression detection of stress-responsive genes

Two-week-old seedlings of transgenic plants together with WT plants (*N* = 5) were treated with 16% PEG 8000 for 24 h. The expression levels of three downstream drought-stress-related genes, *DREB2A*, *P5CS1*, and *RD29A* were detected using RT-qPCR with specific primers, as described above (Table 1). The *Arabidopsis* β-actin was used as internal control. cDNA product was used as template for 40 cycles of 95 °C 2 min, 95 °C 30 s, 59 °C 30 s, 72 °C 30 s. The 2^-△△Ct^ method was used for quantitative analysis.

### Statistical analyses

Statistical analysis was conducted using SPSS version 17.0 (SPSS Inc., Chicago, IL). All data were expressed as mean ± standard deviation (SD). Comparison between different groups was tested by one-way ANOVA, followed by student’s t-test. A *p*-value less than 0.05 was considered significantly different.

## Results

### Cloning of three *TaMYBsm3* homologue genes

**Fig. 1:**
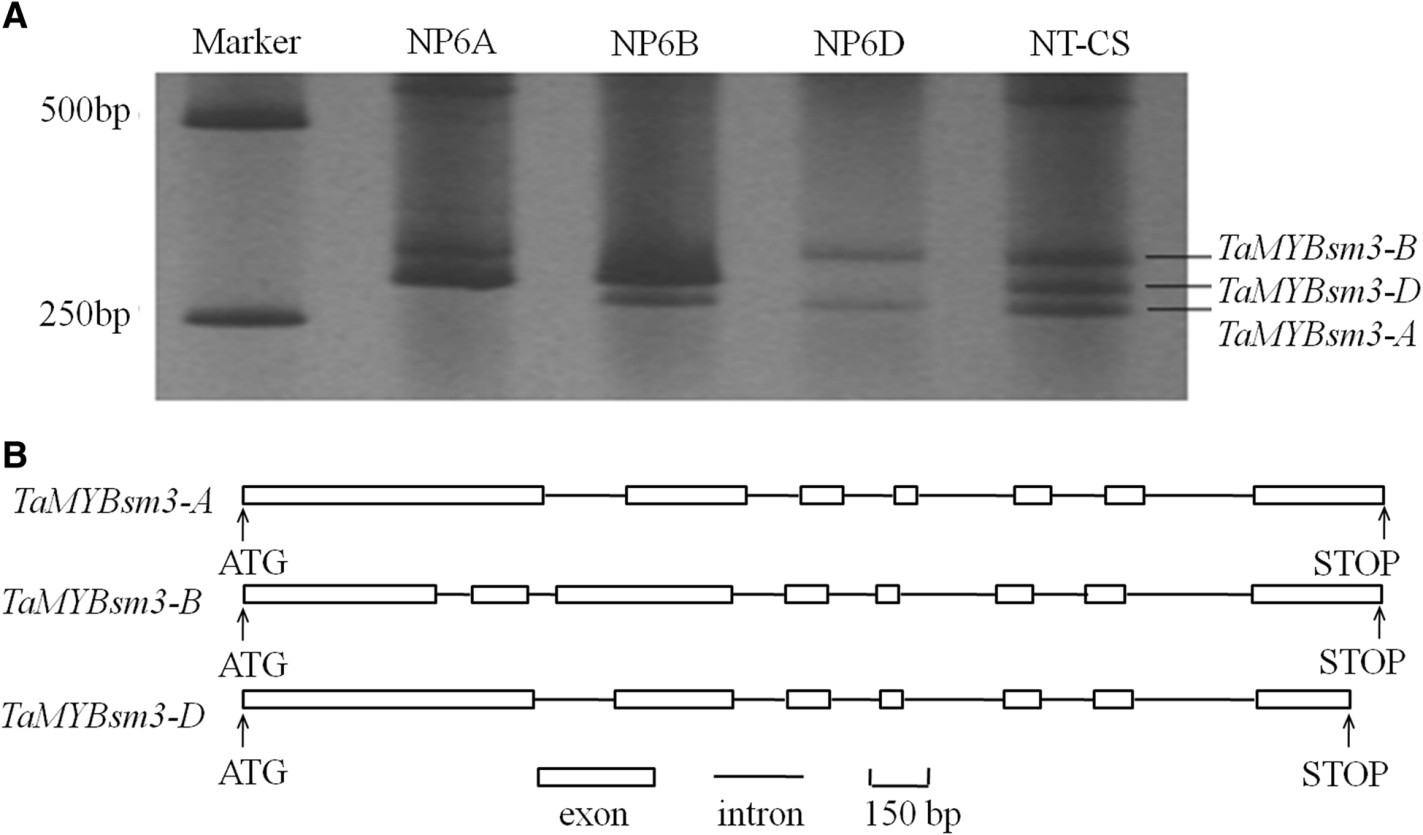
Cloning of three *TaMYBsm3* homologue genes (*TaMYBsm3*-*A*, *TaMYBsm3*-*B,* and *TaMYBsm3*-*D*). **a**, The amplification of *TaMYBsm3* homologue genes; **b**, The structure of *TaMYBsm3* homologue genes. NP6A, NP6B, and NP6D represented three full-length genomic DNA sequences containing *TaMYBsm3* homologue genes cloned from bacterial artificial chromosomal (BAC) libraries of *Triticum aestivum* cultivar Shimai 15. NT-CS represented *TaMYBsm3*-*A*, *TaMYBsm3*-*B,* and *TaMYBsm3*-*D* cloned from null-tetrasomic stocks of Chinese Spring

Three full-length genomic DNA sequences containing *TaMYBsm3* homologue genes (*NP6A*, *NP6B*, and *NP6D*) were cloned in BAC libraries of the *T. aestivum* cultivar Shimai 15. These three genes were mapped on chromosomes 6A, 6B, and 6D in NT-CS, and named as *TaMYBsm3*-*A*, *TaMYBsm3*-*B*, and *TaMYBsm3*-*D*, respectively (Fig. 1a). When blasting with the genomic sequence of NT-CS (https://plants.ensembl.org/Triticum_aestivum/Info/Index), *TaMYBsm3*-*A*, *TaMYBsm3*-*B*, and *TaMYBsm3*-*D* were highly consistent with the sequences on chromosomes 6AS, 6BS, and 6DS, respectively. Sequence analysis showed that *TaMYBsm3*-*A* and *TaMYBsm3*-*D* had the same gene structures with 7 exons and 6 introns, and *TaMYBsm3*-*B* had 8 exons and 7 introns. The ORFs of *TaMYBsm2*-*A*, *TaMYBsm3*-*B*, and *TaMYBsm3*-*D* were 1248 bp, 1152 bp, and 1161 bp in length, encoding 415, 383, and 386 amino acids, respectively (Fig. 1b). *TaMYBsm3*-*A*, *TaMYBsm3*-*B*, and *TaMYBsm3*-*D* shared 90.4–92.9% homology at the nucleotide level and 86.1–93.0% homology at the amino acid level.

### Phylogenetic analysis and multiple alignments of MYBsm3-like proteins

**Fig. 2:**
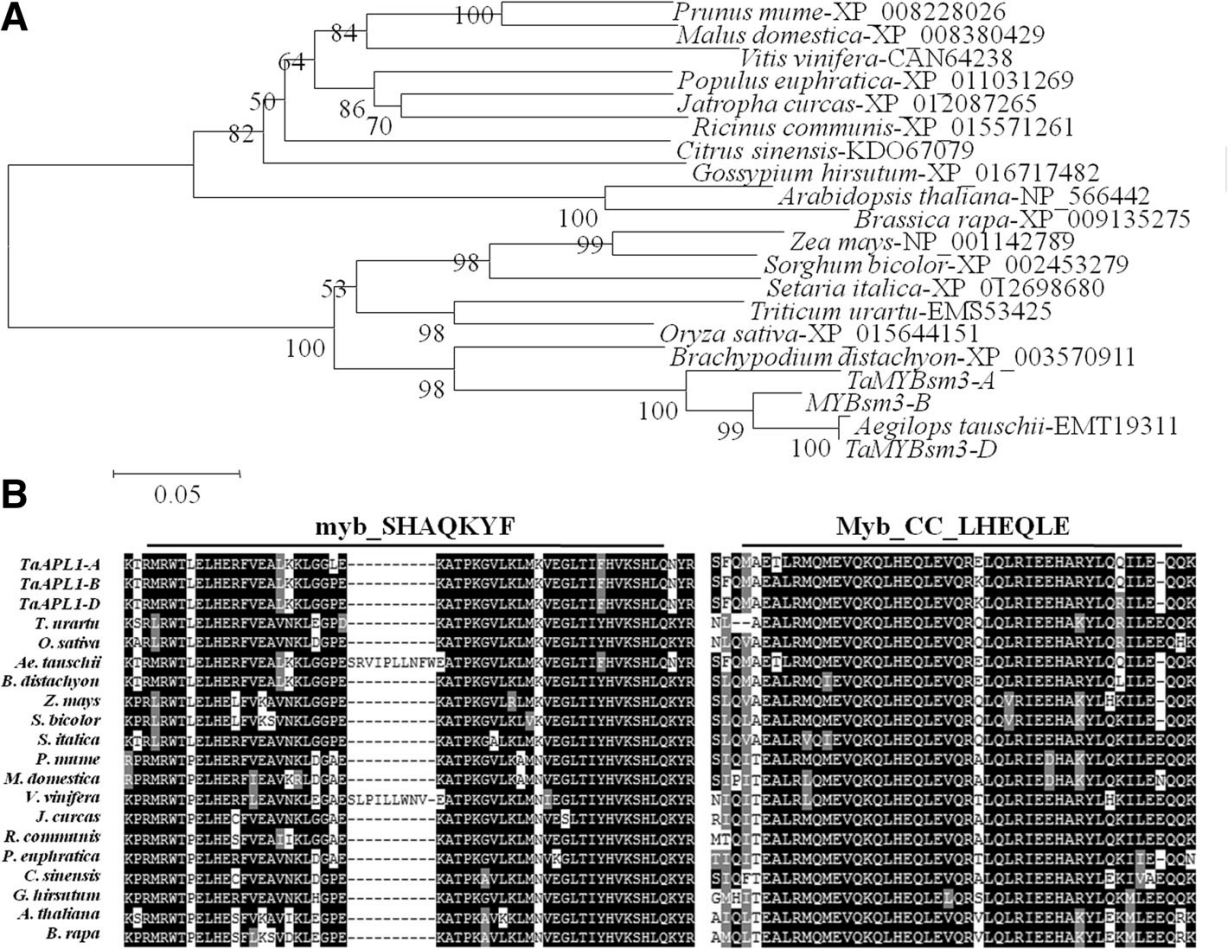
Phylogenetic analysis and multiple alignments of 20 MYBsm3-like proteins from different plants. **a**, Phylogenetic tree; **b**, Multiple alignments. A highly conserved MYB DNA-binding domain (myb_SHAQKYF) and a highly conserved CC domain (Myb_CC_LHEQLE) were observed in MYBsm3-like proteins. Black shadow represented the conserved residues

A total of 20 MYBsm3-like proteins isolated from different plants were used for phylogenetic analysis. As shown in Fig. 2a, MYBsm3-like proteins were divided into two divergent branches, including monocot and dicot. *TaMYBsm3*-*A*, *TaMYBsm3*-*B,* and *TaMYBsm3*-*D* were evolutionarily close to *AeMYBsm3* in *Aegilops tauschii* (Fig. 2a)*.* Multiple alignments showed that MYBsm3-like proteins contained a highly conserved MYB DNA-binding domain (myb_SHAQKYF) and a highly conserved CC domain (Myb_CC_LHEQLE) (Fig. 2b).

### Transcriptional activity and subcellular localization of *TaMYBsm3-D*

**Fig. 3:**
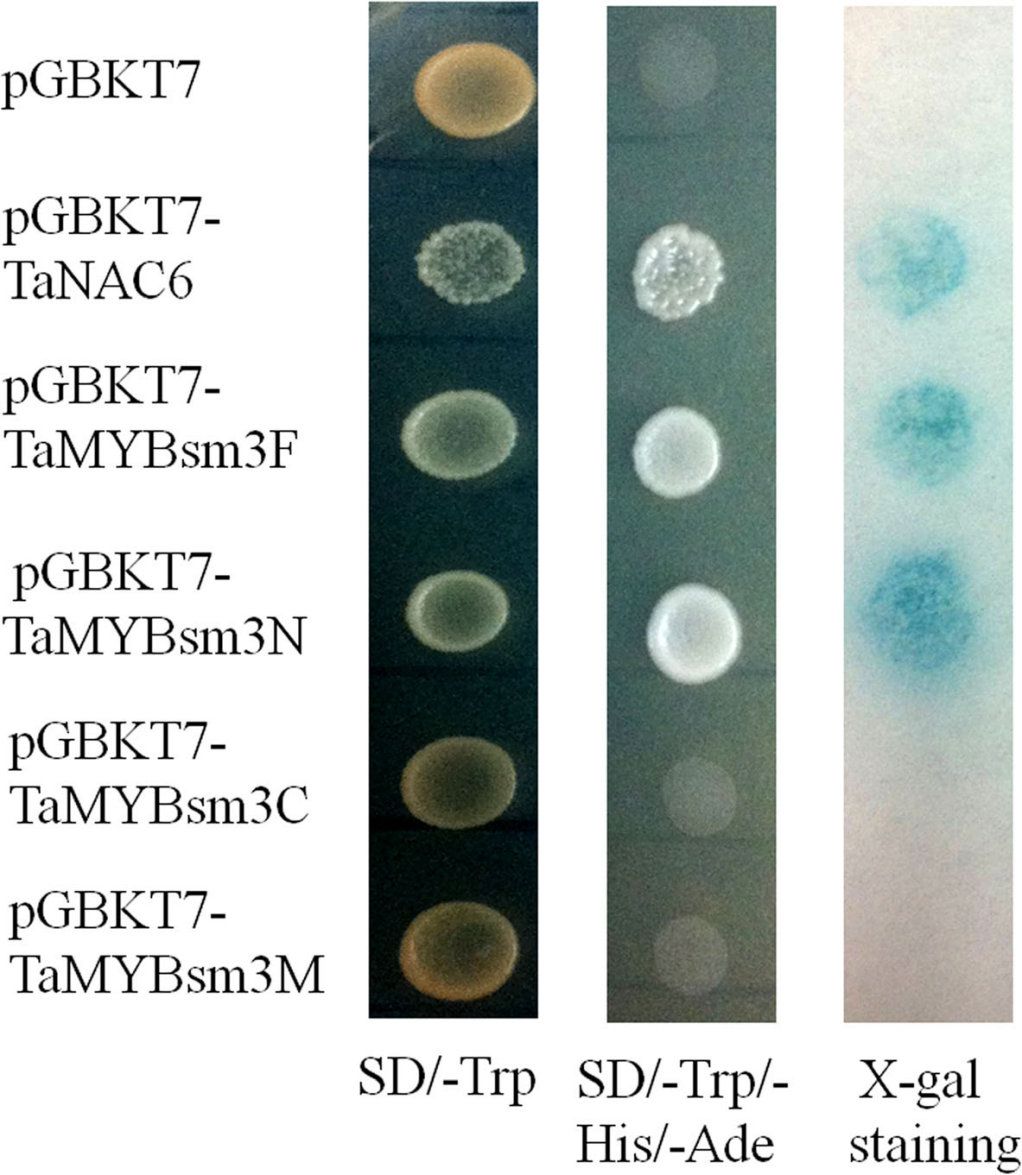
Transactivation assay of *TaMYBsm3-D* in yeast cells. The full-length cDNA (TaMYBsm3F), N-terminal region (TaMYBsm3N), C-terminal region (TaMYB-sm3C), and myb-SHAQKYF region (TaMYBsm3M) were integrated into a pGBKT7 plasmid. β-galactosidase activity was detected by X-gal staining in SD/−Trp/−His/−Ade plate

The transcriptional activity of *TaMYBsm3-D* was analyzed in yeast. The full-length cDNA (TaMYBsm3F), N-terminal region (TaMYBsm3N), C-terminal region (TaMYB-sm3C), and myb-SHAQKYF region (TaMYBsm3M) were transformed into yeast strain Y190. As shown in Fig. 3, the activity of β-galactosidase was found in yeast cells carrying TaMYBsm3F and TaMYBsm3N, but not in yeast cells carrying TaMYBsm3C and TaMYBsm3M (Fig. 3). This result indicated that the N-terminal region of *TaMYBsm3* was a transcriptional activation domain.

**Fig. 4:**
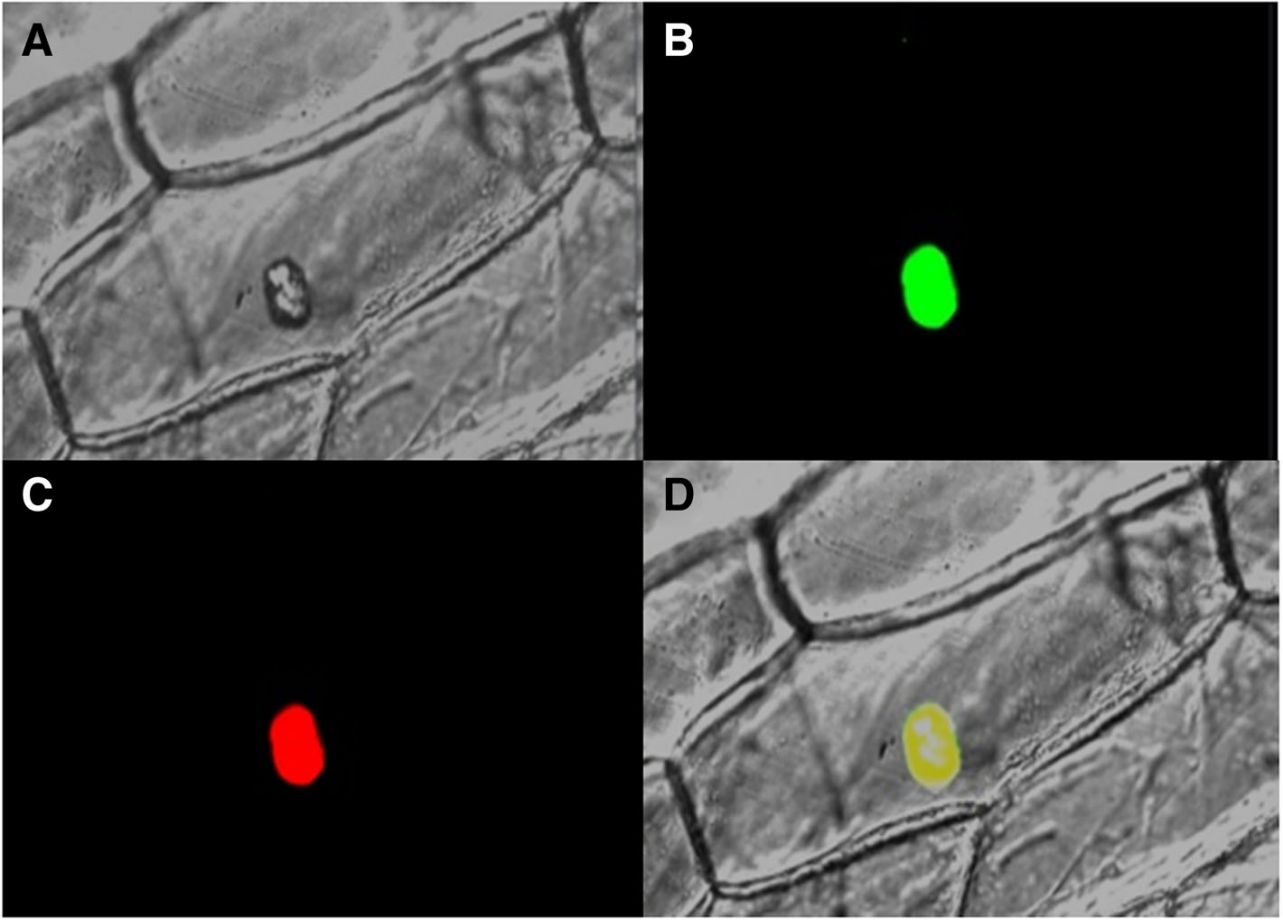
Subcellular localization of TaMYBsm3-D in tobacco epidermal cells. **a**, Bright field; **b**, Green fluorescence of GFP; **c**, Red fluorescence of DAPI; **d**, Merged image of **b** and **c**

To examine the subcellular localization of TaMYBsm3-D, the full-length cDNA sequence of *TaMYBsm3-D* was integrated into pCAMBIA1300-sGFP. The fluorescent signal of GFP was found in the nucleus of transformed tobacco epidermal cells. This phenomenon indicated that TaMYBsm3-D was localized in the nucleus (Fig. 4).

### Expression pattern of *TaMYBsm3* homologue genes in wheat

**Fig. 5:**
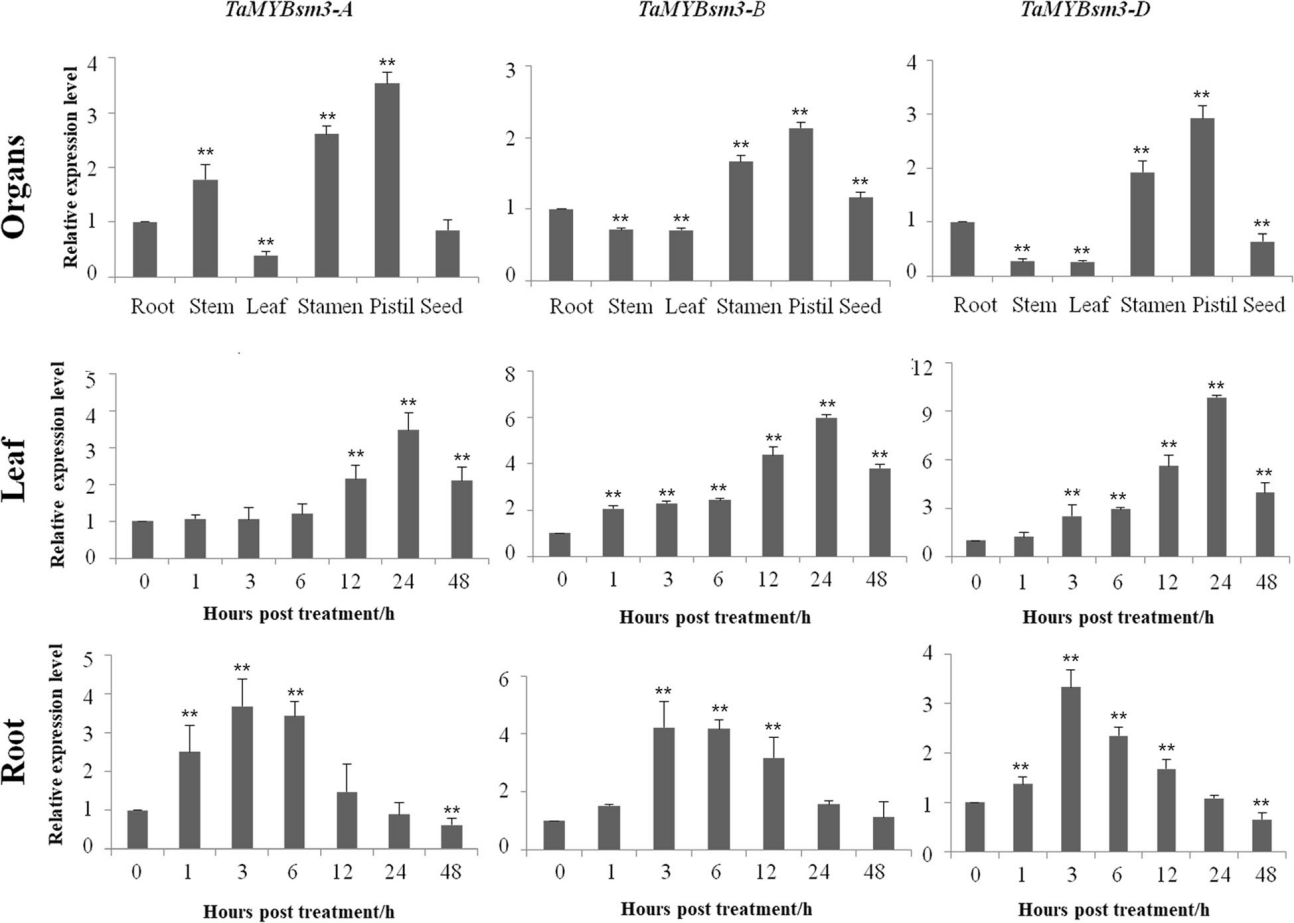
Expression levels of *TaMYBsm3-A*, *TaMYBsm3-B* and *TaMYBsm3-D* in different tissues of wheat under normal conditions, and in the leaf and root of wheat under drought stress (16% PEG8000). The asterisks indicate the statistically significant differences which were determined through student’s t-tests (*P < 0.05, **P < 0.01)

RT-qPCR was used to study the expression pattern of three T*aMYBsm3* homologue genes in wheat. *TaMYBsm3*-*A*, *TaMYBsm3*-*B,* and *TaMYBsm3*-*D* exhibited a consistent expression pattern in wheat. As shown in Fig. 5, T*aMYBsm3* genes were ubiquitously expressed in the root, stem, leaf, stamen, pistil, and seed, and was especially highly expressed in the stamen and pistil. Because of the treatment with 16% PEG8000, the expression of T*aMYBsm3* genes was significantly increased in the leaf with a peak at 24 h, and in the root with a peak at 3 h (Fig. 5).

### Overexpression of *TaMYBsm3-D* improved the drought tolerance of transgenic plants

**Fig. 6:**
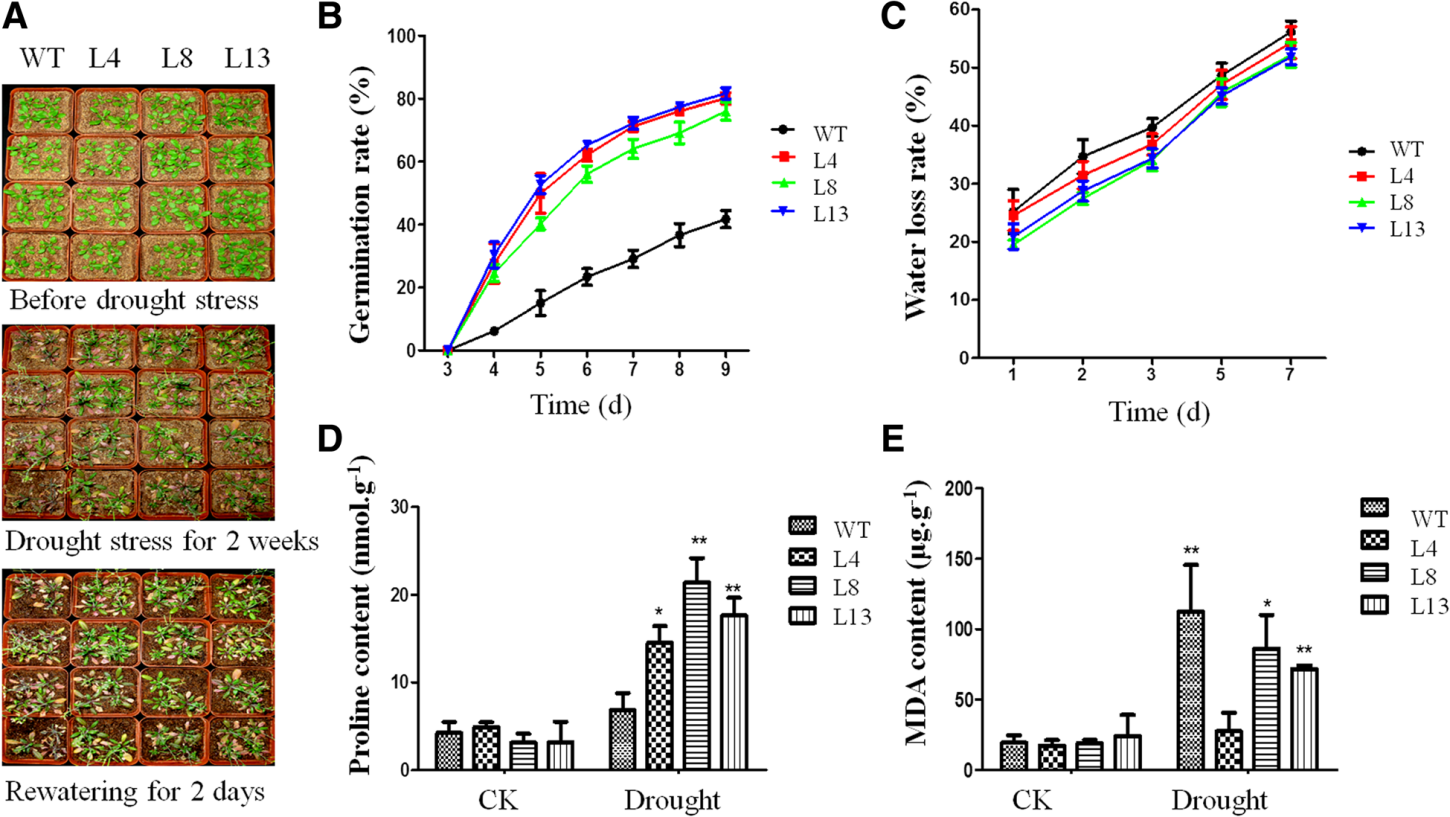
Drought stress response of transgenic *Arabidopsis* plants with *TaMYBsm3-D*. **a**, Phenotypes of transgenic plants before and after drought stress; **b**, Germination rate; **c**, Water loss rate; **d**, Proline content; E, MDA content. *, vs. wild type (WT) at P < 0.05; **, vs. WT at *P* < 0.001

To reveal the drought tolerance ability of *TaMYBsm3-D*, *TaMYBsm3-D* was transformed into *Arabidopsis*. After 4 weeks of growth under normal conditions, both the WT plants and the transgenic lines (L4, L8, and L13) exhibited normal phenotypes and growth status. After 2 weeks of water deprivation, severe wilting symptoms were observed in the WT, whereas the wilting symptoms were mild in transgenic lines. After 2 days of re-watering, most WT plants died, whereas some transgenic plants recovered to normal growth status (Fig. 6a). The germination rates of transgenic plants were higher than that in WT plants under drought stress (*P* < 0.01) (Fig. 6b). The water loss rates were significantly lower in transgenic plants compared with in WT plants under normal conditions (*P* < 0.05) (Fig. 6c). Furthermore, significantly higher proline and lower MDA contents were revealed in transgenic plants compared with that of the WT (P < 0.05) (Fig. 6d, e). Taken together, the above results indicated that the drought tolerance of transgenic *Arabidopsis* was improved by overexpressed *TaMYBsm3-D*.

### Abiotic stress-responsive genes were upregulated in *TaMYBsm3-D* transgenic *Arabidopsis*

**Fig. 7:**
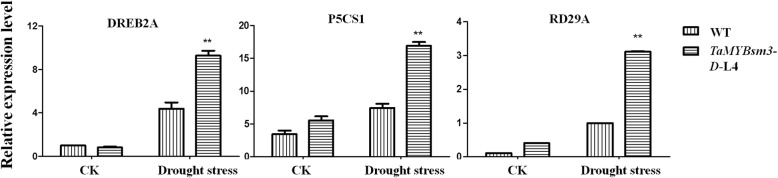
The expression of three abiotic stress-responsive genes (*DREB2A*, *P5CS1*, and *RD29A*). CK, normal condition. *, vs. WT at P < 0.05; **, vs. wild type (WT) at P < 0.01

To reveal the molecular mechanisms related to the drought response of *TaMYBsm3-D* transgenic plants, the expression levels of *DREB2A*, *P5CS1*, and *RD29A* (three abiotic stress-responsive genes) were detected. As shown in Fig. 7, the expression levels of *DREB2A*, *P5CS1*, and *RD29A* were significantly higher in transgenic plants than in WT plants (P < 0.01) (Fig. 7). The results indicated that *TaMYBsm3-D* may enhance the drought tolerance through up-regulating abiotic stress-responsive genes.

## Discussion

Drought is a major environmental stress which limits the productivity of wheat. Searching for novel genes involved in drought tolerance has become a hot topic in breeding [29]. Recently, some MYB-CC genes have been described to be related to Pi availability, but their role in drought stress tolerance of plants has not been reported.

The *PHR1*-like genes, *TaPHR1* genes, were mapped on chromosomes 7A, 4B, 4D and involved in Pi signaling in bread wheat [22]. In this study, three *TaMYBsm3* homologue genes with potential drought resistance ability were isolated from *T. aestivum* Shimai 15 by screening the BAC library. The three genes were mapped on chromosomes 6A, 6B, and 6D in wheat, and named *TaMYBsm3-A*, *TaMYBsm3*-*B*, and *TaMYBsm3*-*D*, respectively. *TaPHR1* and *TaMYBsm3* share 32.1–33.7% identity at the nucleotide level and 23.1–26.7% identity at amino acid level, but their two motifs, myb_SHAQKYF and coiled-coil, are highly conserved. The results indicate that *TaPHR1* and *TaMYBsm3* are members of MYB-CC TF family, but they are strongly divergent at the nucleotide and amino acid levels, which may be associated with their diverse functions.

*TaMYBsm3* belongs to MYB-CC TF, containing a conserved SHAQKYF motifMYB DNA-binding domain at the N-terminal and a conserved CC domain at the C-terminal. The two motifs of plant MYBsm3 homologues exhibited significant conservation and showed no significant difference between dicot and monocot plants [30]. Our further assays showed that *TaMYBsm3-D* was localized in the nucleus, and presented a transactivation activity of a GAL4-containing reporter gene, indicating the predicted role of *TaMYBsm3-D* as a TF. The subcellular localization of *TaMYBsm3-D* was consistent with most known *MYB* genes from diverse species, such as *AtMYB26* [31], *OsMYB48–1* [13], *TiMYB2R-1* [32], and *TaMYB80* [33]. The MYB DNA-binding domain at the N-terminal directly contributed to the transcriptional activation of *TaMYBsm3-D.*

In this study, the involvement of *TaMYBsm3* to drought stress was firstly analyzed by RT-qPCR in wheat. The *TaMYBsm3* gene was induced by 16% PEG8000, but presented different expression profiles in leaves and roots of bread wheat. The expression of T*aMYBsm3* genes was significantly increased in the leaf with a peak at 24 h, while in the root with a peak at 3 h, which indicated that *TaMYBsm3* genes were sensitive to drought stress in leaves and roots, and the response of T*aMYBsm3* was higher in leaves than in roots. Then, the drought resistance ability of *TaMYBsm3-D* was further evaluated using *TaMYBsm3-D* transgenic *Arabidopsis* plants. The result showed that the transgenic plants exhibited milder wilting symptoms than WT plants under drought stress, and could recover to normal growth status after re-watering. Furthermore, significantly lower water loss rates were observed in transgenic plants comparing with that of WT plants. These phenomena illustrated that the transgenic plants had greater drought tolerance than WT plants. Specially, transgenic plants presented significantly higher germination rates WT plants than WT plants. For most seeds, germination begins with the imbibition of water, which generally included three processes: rapid initial water uptake, a plateau phase with little change in water content, and an increased water content coincident with radicle growth [34]. Although moisture is an important factor that determines the germination rate, there are many other factors that affact the germination rate, such as temperature, oxygen, light, and some intrinsic factors. Thus, the higher germination rate of transgenic plants may be related to other mechanisms. The drought resistance characteristics of *TaMYBsm3-D* are consistent with several known drought resistant MYB genes, such as *AtMYB96* [35], *PbrMYB21* [12], *OsMYB48–1* [13], and *TaODORANT1* [14].

Proline and MDA are important physiological indicators of drought stress [36, 37]. Drought stress can damage the membrane of plant cells and induce the production of active oxygen in plant cells, leading to membrane lipid peroxidation [38]. MDA is known as a biomarker of lipid peroxidation [36]. Furthermore, proline acts as an osmoprotectant in plants subjected to drought stress [39]. In this study, significantly higher proline and lower MDA contents were revealed in *TaMYBsm3*-D transgenic *Arabidopsis* under drought stress compared with that of WT plants. These results suggest that the drought resistance ability of *TaMYBsm3-D* is associated with relieved lipid peroxidation and stable osmotic pressure.

Currently, the transcriptional regulatory networks of abiotic stress responses have been deeply studied in *Arabidopsis*. Some stress-responsive genes provide important information regarding the mechanism of *TaMYBsm3-D* involved in the drought stress response. In our study, *P5CS1*, *DREB2A*, and *RD29A*, three downstream genes, were upregulated significantly in transgenic plants under drought stress. *DREB2A* is TF associated with drought and high-salinity stress responses [40]. It has been reported that up-regulated *DREB2A* enhances drought tolerance by activating stress-inducible genes [41]. In *Arabidopsis*, *DREB2A* could be induced by high-salt stress or dehydration (Liu et al., 1998; Nakashima et al., 2000; Sakuma et al., 2002). The up-regulated *DREB2A* enhances the tolerance of *TaMYBsm3-D* transgenic plants to drought stress by activating stress-induced genes. P5CS is known to be a rate-limiting enzyme in proline biosynthesis, mutations of which result in decreased salt tolerance in transgenic plants (Székely et al., 2008). The overexpression of *P5CS* promotes the drought tolerance of tobacco [42, 43], whereas silencing of *P5CS1* inhibited the drought tolerance through suppressing proline synthesis and reactive oxygen species accumulation [44]. The accumulation of proline induced by *P5CS* can enhance the drought tolerance of *TaMYBsm3-D* transgenic plants. Additionally, *RD29A* is important in the ABA-related response to drought through regulating the osmotic potential [45]. Desiccation induced *RD29A* with two-step kinetics, which indicated that *RD29A* had two or more cis-acting elements, one associated with the ABA-associated response to desiccation, while the other induced by osmotic potential changes (Yamaguchi-Shinozaki and Shinozaki, 1993, Shinozaki and Yamaguchi- Shinozaki, 2007; Hirayama and Shinozaki, 2010). Under drought stress, overexpression of *TaMYBsm3-D* increased the expression level of *DREB2A* in transgenic *Arabidopsis* and their drought tolerances were enhanced by activating some stress-inducible genes. Due to the upregulation of *P5CS1*, proline accumulated in *TaMYBsm3* transgenic *Arabidopsis*, which increased the drought tolerance of the transgenic plants. In summary, we hypothesize that TaMYBsm3-D may function in drought tolerance through increasing the expression of abiotic stress-responsive genes, including *P5CS1*, *DREB2A*, and *RD29A*. The present study only analyzed the transcriptional regulation of genes to explain the stress response at protein level. Further study is needed to elucidate the regulation mechanism at translational level. Additionally, functional verification of the effect of *TaMYBsm3* genes on drought tolerance is still limited to *Arabidopsis.* Further research on the roles of *TaMYBsm3* genes in wheat and regulation mechanisms at the translational level are still needed.

## Conclusion

In conclusion, the *TaMYBsm3* genes are MYB-CC type TF genes. Overexpression of *TaMYBsm3*-*D* improves the drought tolerance of transgenic *Arabidopsis* through up-regulating *P5CS1*, *DREB2A*, and *RD29A.*

## Abbreviations

BAC: Bacterial artificial chromosomal
CC: Coiled-coil
EST: Expressed sequence tags
MDA: Malondialdehyde
NP: Nested PCR primers
NRF: Nulliso open reading frame
NT-CS: Nullisomic tetrasomic line of Chinese Spring
ORF: Open reading frame
PEG: Polyethylene glycol
ROS: Reactive oxygen species
RT-qPCR: Real-time quantitative PCR
SD: Standard deviation
TFs: Transcription factors
WT: Wild type

## References

[1] Xiao W, Vignjevic M, Dong J, Jacobsen S, Wollenweber B (2014). Improved tolerance to drought stress after anthesis due to priming before anthesis in wheat (Triticum aestivum L.) var. Vinjett. J Exp Bot.

[2] Lenka SK, Katiyar A, Chinnusamy V, Bansal KC (2011). Comparative analysis of drought-responsive transcriptome in Indica rice genotypes with contrasting drought tolerance. Plant Biotechnol J.

[3] Shinozaki K, Yamaguchi-Shinozaki K, Shinozaki K, Yamaguchi-Shinozaki K (2007). Gene networks involved in drought stress response and tolerance. J Exp Bot.

[4] Hussain SS, Kayani MA, Amjad M (2011). Transcription factors as tools to engineer enhanced drought stress tolerance in plants. Biotechnol Prog.

[5] Golldack D, Lüking I, Yang O (2011). Plant tolerance to drought and salinity: stress regulating transcription factors and their functional significance in the cellular transcriptional network. Plant Cell Rep.

[6] Zhang L, Zhao G, Xia C, Jia J, Liu X, Kong X (2012). A wheat R2R3-MYB gene, TaMYB30-B, improves drought stress tolerance in transgenic Arabidopsis. J Exp Bot.

[7] Chen Y, Yang X, He K, Liu M, Li J, Gao Z, Lin Z, Zhang Y, Wang X, Qiu X (2006). The MYB transcription factor superfamily of Arabidopsis: expression analysis and phylogenetic comparison with the Rice MYB family. Plant Mol Biol.

[8] Riechmann JL, Heard J, Martin G, Reuber L, Jiang CZ, Keddie J, Adam L, Pineda O, Ratcliffe OJ, Samaha RR (2000). Arabidopsis transcription factors: genome-wide comparative analysis among eukaryotes. Science.

[9] Antje F, Katja M, Braun EL, Erich G (2011). Evolutionary and comparative analysis of MYB and bHLH plant transcription factors. Plant J Cell Mol Biol.

[10] Dubos C, Stracke R, Grotewold E, Weisshaar B, Martin C, Lepiniec L (2010). MYB transcription factors in Arabidopsis. Trends Plant Sci.

[11] Hichri I, Barrieu F, Bogs J, Kappel C, Delrot S, Lauvergeat V (2011). Recent advances in the transcriptional regulation of the flavonoid biosynthetic pathway. J Exp Bot.

[12] Li K, Xing C, Yao Z, Huang X (2017). PbrMYB21, a novel MYB protein of Pyrus betulaefolia, functions in drought tolerance and modulates polyamine levels by regulating arginine decarboxylase gene. Plant Biotechnol J.

[13] Xiong H, Li J, Liu P, Duan J, Zhao Y, Guo X, Li Y, Zhang H, Ali J, Li Z (2014). Overexpression of OsMYB48-1 , a novel MYB-related transcription factor, enhances drought and salinity tolerance in Rice. PLoS One.

[14] Wei Q, Luo Q, Wang R, Zhang F, He Y, Zhang Y, Qiu D, Li K, Chang J, Yang G. A wheat R2R3-type MYB transcription factor TaODORANT1 positively regulates drought and salt stress responses in transgenic tobacco plants. Front Plant Sci. 2017;8. 10.3389/fpls.2017.01374 PMC5550715 28848578

[15] Seo P. J., Lee S. B., Suh M. C., Park M.-J., Go Y. S., Park C.-M. (2011). The MYB96 Transcription Factor Regulates Cuticular Wax Biosynthesis under Drought Conditions in Arabidopsis. The Plant Cell.

[16] Zhao Y, Cheng X, Liu X, Wu H, Bi H, Xu H. The wheat MYB transcription factor TaMYB31 is involved in drought stress responses in Arabidopsis. Front Plant Sci. 2018;9:1–12. 10.3389/fpls.2018.01426 PMC6172359 30323824

[17] Fujita Y, Fujita M, Shinozaki K, Yamaguchi-Shinozaki K (2011). ABA-mediated transcriptional regulation in response to osmotic stress in plants. J Plant Res.

[18] Seiler C, Harshavardhan VT, Rajesh K, Reddy PS, Strickert M, Rolletschek H, Scholz U, Wobus U, Sreenivasulu N (2011). ABA biosynthesis and degradation contributing to ABA homeostasis during barley seed development under control and terminal drought-stress conditions. J Exp Bot.

[19] Wykoff DD, Grossman AR, Weeks DP, Usuda H, Shimogawara K (1999). Psr1, a nuclear localized protein that regulates phosphorus metabolism in Chlamydomonas. Proc Natl Acad Sci.

[20] Xue Y-B, Xiao B-X, Zhu S-N, Mo X-H, Liang C-Y, Tian J, Liao H, Miriam G (2017). GmPHR25, a GmPHR member up-regulated by phosphate starvation, controls phosphate homeostasis in soybean. J Exp Bot.

[21] Ruan W, Guo M, Wu P, Yi K (2017). Phosphate starvation induced OsPHR4 mediates pi-signaling and homeostasis in rice. Plant Mol Biol.

[22] Wang J, Sun J, Miao J, Guo J, Shi Z, He M, Chen Y, Zhao X, Li B, Han F (2013). A phosphate starvation response regulator Ta-PHR1 is involved in phosphate signalling and increases grain yield in wheat. Ann Bot.

[23] Zhang L, Zhao G, Jia J, Liu X, Kong X (2012). Molecular characterization of 60 isolated wheat MYB genes and analysis of their expression during abiotic stress. J Exp Bot.

[24] Meng-jun L, Yu Y, Xin-na G, Xiao G, Ming-qi H, Zhan-liang S, Jin-kao G (2014). Construction and characterization of BAC library from common wheat Shimai15. J Triticeae Crops.

[25] Park J, Mi JK, Su JJ, Mi CS (2009). Identification of a novel transcription factor, AtBSD1, containing a BSD domain in Arabidopsis thaliana. J Plant Biol.

[26] Breeden L, Nasmyth K (1985). Regulation of the yeast HO gene. Cold Spring Harb Symp Quant Biol.

[27] Clough SJ, Bent AF (1998). Floral dip: a simplified method forAgrobacterium-mediated transformation ofArabidopsis thaliana. Plant J Cell Mol Biol.

[28] Draper HH, Hadley M (1990). Malondialdehyde determination as index of lipid peroxidation. Methods Enzymol.

[29] Mohammadi Reza (2018). Breeding for increased drought tolerance in wheat: a review. Crop and Pasture Science.

[30] Rubio V, Linhares F, Solano R, Martín AC, Iglesias J, Leyva A, Paz-Ares J (2001). A conserved MYB transcription factor involved in phosphate starvation signaling both in vascular plants and in unicellular algae. Genes Dev.

[31] Yang C, Xu Z, Song J, Conner K, Vizcay BG, Wilson ZA (2007). Arabidopsis MYB26/MALE STERILE35 regulates secondary thickening in the endothecium and is essential for anther dehiscence. Plant Cell.

[32] Liu X, Yang L, Zhou X, Zhou M, Lu Y, Ma L, Ma H, Zhang Z (2013). Transgenic wheat expressing Thinopyrum intermedium MYB transcription factor TiMYB2R-1 shows enhanced resistance to the take-all disease. J Exp Bot.

[33] Zhao Y, Tian X, Wang F, Zhang L, Xin M, Hu Z, Yao Y, Ni Z, Sun Q, Peng H (2017). Characterization of wheat MYB genes responsive to high temperatures. BMC Plant Biol.

[34] Bradford KJ (1990). A water relations analysis of seed germination rates. Plant Physiol.

[35] Saetbuyl L, Hyojin K, Ryeojin K, Michung S (2014). Overexpression of Arabidopsis MYB96 confers drought resistance in Camelina sativa via cuticular wax accumulation. Plant Cell Rep.

[36] Roychoudhury A, Roy C, Sengupta DN (2007). Transgenic tobacco plants overexpressing the heterologous lea gene Rab16A from rice during high salt and water deficit display enhanced tolerance to salinity stress. Plant Cell Rep.

[37] Zhang L, Zhang L, Xia C, Zhao G, Jia J, Kong X (2016). The novel wheat transcription factor TaNAC47 enhances multiple abiotic stress tolerances in transgenic plants. Front Plant Sci.

[38] Mirzaee M, Moieni A, Ghanati F (2013). Effects of drought stress on the lipid peroxidation and antioxidant enzyme activities in two canola (Brassica napus L.) cultivars. J Agric Sci Technol.

[39] Yamada M, Morishita H, Urano K, Shiozaki N, Yamaguchi-Shinozaki K, Shinozaki K, Yoshiba Y (2005). Effects of free proline accumulation in petunias under drought stress. J Exp Bot.

[40] Nakashima K, Shinwari ZK, Sakuma Y, Seki M, Miura S, Shinozaki K, Yamaguchi-Shinozaki K (2000). Organization and expression of two Arabidopsis DREB2 genes encoding DRE-binding proteins involved in dehydration- and high-salinity-responsive gene expression. Plant Mol Biol.

[41] Sakuma Y, Maruyama K, Osakabe Y, Qin F, Seki M, Shinozaki K, Yamaguchi-Shinozaki K (2006). Functional analysis of an Arabidopsis transcription factor, DREB2A, involved in drought-responsive gene expression. Plant Cell.

[42] Yamchi A, Jazii FR, Ghobadi C, Mousavi A (2005). Increasing of tolerance to osmotic stresses in tobacco Nicotiana tabacum cv. Xanthi through overexpression of p5cs gene. J Sci Technol Agric Nat Resour.

[43] Kishor P, Hong Z, Miao GH, Hu C, Verma D (1995). Overexpression of [delta]-Pyrroline-5-carboxylate synthetase increases proline production and confers Osmotolerance in transgenic plants. Plant Physiol.

[44] Székely G, Abrahám E, Cséplo A, Rigó G, Zsigmond L, Csiszár J, Ayaydin F, Strizhov N, Jásik J, Schmelzer E (2008). Duplicated P5CS genes of Arabidopsis play distinct roles in stress regulation and developmental control of proline biosynthesis. Plant J.

[45] Hirayama T, Shinozaki K (2010). Research on plant abiotic stress responses in the post-genome era: past, present and future. Plant J Cell Mol Biol.

